# The effect of motivational interviewing on internet addiction and digital game addiction in adolescents: a randomized controlled trial

**DOI:** 10.1007/s00431-026-06993-5

**Published:** 2026-05-02

**Authors:** Semra Seyhan Şahin, Sultan Ayaz-Alkaya

**Affiliations:** 1 Semra ve Vefa Küçük Faculty of Health Sciences, Nevşehir Hacı Bektas Veli Universtiy, Nevşehir, Turkey; 2 Faculty of Nursing, Gazi University, Emek Bişkek Street, 6th street, No. 2, Ankara, Turkey

**Keywords:** Adolescent, Internet addiction, Motivational interviewing, Digital game addiction

## Abstract

The purpose of this study was to measure the effect of motivational interviewing on both reducing internet addiction and digital game addiction in adolescents. A parallel-group randomised controlled trial was adopted. The study population consisted of ninth-grade (14–15 years of age) high school students in a city in Turkiye. The study was completed by 88 participants (experimental: 44; control: 44). The data were collected using a Personal Information Form, the Young Internet Addiction Test, and the Digital Game Addiction Scale. The experimental group received a preparatory session and five weekly motivational interviewing sessions. Instruments were administered to both groups before (pre-test) and after the intervention (post-test), and at follow-up tests 3 and 6 months after the final session. The data were analysed using the two-way mixed design and the Bonferroni Comparison Test. The mean scores of internet addiction and digital game addiction decreased significantly after the motivational interviewing in the experimental group compared to the control group (*p* < 0.001) in both the post-test and follow-up tests.

*Conclusion*: The present study concluded that motivational interviewing may be associated with reductions in mitigating symptoms of internet addiction and digital game addiction behaviours among adolescents. Motivational interviewing could be implemented to reduce internet addiction and digital game addiction behaviours.

*Trial registration*: The study was registered on a clinical trial database (NCT06721702). The study started on December 11, 2023 (actual date on which the first participant was enrolled).

**Table:** 

**What is Known:** • *Internet addiction and digital game addiction are two increasingly important problems among adolescents.* • *Digital games and online activities negatively affect adolescents’ physical, social, and psychological health.*	
**What is New:** • *Motivational interviewing was an effective technique to reduce online gaming and internet addiction.* • *A motivational interviewing program comprising at least six sessions could be implemented to promote behavioural change in adolescents.*

**What is Known:**

• *Internet addiction and digital game addiction are two increasingly important problems among adolescents.*

• *Digital games and online activities negatively affect adolescents’ physical, social, and psychological health.*

**What is New:**

• *Motivational interviewing was an effective technique to reduce online gaming and internet addiction.*

• *A motivational interviewing program comprising at least six sessions could be implemented to promote behavioural change in adolescents.*

## Introduction

Adolescence is a developmentally risky period. During this time, the prefrontal cortex, responsible for executive functions, is not fully mature, impulse control is limited, and the reward system is dominant, all of which contribute to the development of problematic or behavioural addictions [1]. Internet addiction is the excessive and problematic use of the internet and the inability to prevent this behaviour despite the negative consequences in daily life [2]. Adolescents who rapidly adopt current technology and online platforms are especially at risk of internet addiction, in part due to characteristics generally prevalent in young people, such as being curious, a lack of self-control, and an inability to form an identity [3, 4]. Excessive and uncontrolled use of the internet can lead to mental problems such as nomophobia [5], social anxiety [3], depression [6], and internet and game addiction in adolescents [3, 7].

Game addiction is “an excessive and compulsive use of video games that results in social and/or emotional problems; despite these problems, the gamer is unable to control this excessive use” [8]. Several studies have revealed that digital games make up the majority of internet usage, and can lead to addiction [9, 10]. In addition, long-term addiction to digital games can cause symptoms such as anxiety and depression through increased interpersonal loneliness and a decline in the ability to establish and maintain relationships [11].

Internet addiction and digital game addiction, which encapsulate the negative side of rapidly developing digital technologies and increased internet accessibility, are global concerns [12]. Internet addiction and digital game addiction are conceptually related but are not the same [13]. These two behavioural addictions may result from compulsive internet use [14], have similar psychological mechanisms (reward system, cognitive control, and reinforcement) [1], and are associated with similar psychopathological symptoms (including depression, anxiety, attention-deficit or hyperactivity problems, aggression, sleep disturbances, and suicidal ideation) [3, 6, 11, 13]. Therefore, addressing these two issues together is necessary for maintaining mental health in adolescents.

Behavioural addictions, including internet and digital game addictions, are characterized by activation of the reward system, weakening of reinforcement processes, and impaired cognitive control mechanisms [1]. The reward value of a behaviour may depend on both external and internal factors, such as subjective experience and goal achievement [15]. In the behavioural change process, which allows for the systematic linking of motivation and reward mechanisms, individuals can experience the stages of unawareness, awareness, thinking, planning, initiation, ongoing action, and maintenance [16]. Therefore, interventions aimed at behavioural change in adolescents need to address not only the behaviour itself but also the individual’s motivational processes.

Positive results have been observed in studies in which different interventions, such as multidimensional family therapy [12], horticultural therapy [17], and traditional children’s games [18], were applied to reduce internet addiction and digital game addiction in adolescents. However, the prevalence of internet and digital game addiction continues to increase worldwide despite the success of these findings [3, 7, 19]. Therefore, further evidence-based studies are needed to examine the effectiveness of interventions to prevent internet and digital game addiction behaviours [19]. Motivational interviewing can be considered a holistic intervention that addresses behavioural addiction in interaction with neurobiological reward processes, learning mechanisms, and developmental factors [1].

Motivational interviewing is a client-centred psychotherapeutic approach that helps clients with ambivalence make positive decisions, achieve specific and purposeful goals, and increase internal motivation for change [20]. Motivational interviewing activates cognitive control mechanisms and supports change by helping individuals recognise the discrepancy between their values and behaviour [21]. It also strengthens the individual’s belief in their ability to control their behaviour, thereby increasing their sense of self-efficacy [22]. In these respects, the authors suggest that motivational interviewing is a critical psychosocial approach that integrates reward-based learning processes and individual motivational mechanisms underlying internet addiction and digital game addiction in adolescents.

In the literature, there are a limited number of studies examining the effect of motivational interviewing in countering internet addiction [23, 24] and digital game addiction [25, 26]. In these studies, it is proposed that motivational interviewing is effective; however, further studies are needed to definitively establish its efficacy. Considering the interaction between internet addiction and digital game addiction, similar psychological mechanisms, and psychopathological symptoms, it is essential to address both addictions in intervention methods. However, a gap exists in the literature that addresses internet addiction and digital game addiction together and implements a motivational interviewing approach. It is believed that motivational interviewing could contribute to a reduction in addictive behaviours by careful evaluation of the effect of motivational interviewing on both internet addiction and digital game addiction simultaneously.

This study was aimed at measuring the effect of motivational interviewing on reducing internet addiction and digital game addiction in adolescents. The research hypotheses are as follows:

- H_1_: Adolescents who have motivational interviewing have lower levels of internet addiction over time compared to adolescents in the control group.
- H_2_: Adolescents who have motivational interviewing have lower levels of digital game addiction over time compared to adolescents in the control group.

## Methods

### Study design and participants

The study was conducted as a parallel-group randomised controlled trial. The study was registered on a clinical trial database (NCT06721702). The study group consisted of ninth-grade (14–15 years) students (*N* = 290) studying in a high school in a provincial centre in Turkiye. Power analysis was performed based on the internet addiction variable of a previous study [27]. Accordingly, the sample size was calculated to be at least 74 with 95% power, a 5% margin of error, and an effect size (*d*) of 0.85. However, given the possibility of losses, the sample size was increased by approximately 20%, resulting in 90 adolescents included in the study.

Among the adolescents evaluated for study eligibility (*n* = 290), 170 were categorised as not addicted to digital games, 7 were not in the pre-intention or intention stage according to the change stage evaluation form, and 23 refused to participate in the study. The adolescents were assigned to the experimental (*n* = 45) and control (*n* = 45) groups.

The inclusion criteria were (1) being a ninth-grade student, (2) being in the intention or pre-intention stage according to the stage of the change assessment form, (3) being addicted to digital games according to the Digital Game Addiction Scale, (4) having parental approval, and (5) volunteering to participate. The exclusion criterion was the presence of any physical or mental illness. The termination criteria were (1) leaving the study voluntarily, (2) failing to attend at least two motivational interviewing sessions, and (3) an incomplete data collection form.

### Randomisation and prevention of bias

The adolescents included in the study were assigned to the experimental and control groups using stratified randomisation. According to the change stage evaluation form, all adolescents were in the pre-intention stage. Adolescents were divided into two strata by sex (i.e. girls and boys), and assigned to the experimental (*n* = 45) and control (*n* = 45) groups by simple randomisation. To prevent selection bias, a biostatistician independent of the study generated a random sequence of integers and allocated the adolescents to the groups. The data were transferred to the statistical analysis program by participant number. To prevent assessment bias, the post-test and follow-up data were collected by the school’s guidance and counselling teacher, who was outside the research team. The motivational interviewing sessions were conducted according to standard techniques, including open-ended questioning, affirmations, reflective listening, and summarising, to avoid researcher performance bias.

### Instruments

The data were collected using the Personal Information Form, the Young Internet Addiction Test, and the Digital Game Addiction Scale. The Personal Information Form, prepared based on the literature [3, 7], consisted of questions, including age, sex, parental education level, income, family relations, academic achievement, relationship with friends, daily internet use time, owning a smartphone duration, and digital gaming time.

The Young Internet Addiction Test Short Form was developed by Young (1998) for assessing internet addiction [28]. It was shortened by Pawlikowski et al. in 2013 [29]. It is a 5-point Likert-type scale consisting of 12 items and a single factor adapted into Turkish by Kutlu et al. in 2016 [30]. The scores on the scale range from 12 to 60. High scores obtained from the scale indicate a high level of addiction. Cronbach’s alpha coefficient reported in the reliability study was 0.86 in adolescents [30]. In this study, Cronbach’s alpha coefficients of pre-test, post-test, first and second follow-up scores were above an acceptable level (Cronbach’s alpha > 0.80).

The Digital Game Addiction Scale (DGAS) was developed by Lemmens et al. (2009) to assess problematic digital gaming behaviours among adolescents aged 12–18 years [8]. The scale is a seven-item short form of the DGAS-21, consisting of 21 items and seven sub-dimensions. The validity and reliability study of the Turkish version of the scale was examined by Irmak and Erdoğan in 2015 [31]. The scale has a five-point Likert-type, single-factor structure, with scores ranging from 1 (never) to 5 (always) (range: 7–35). The assessment is made using monothetic and polythetic diagnoses. According to the monothetic diagnosis, a person is defined as “game addicted” if they score three (sometimes) or more on all seven items; according to the polythetic diagnosis, a person is defined as “game addicted” if they score 3 (sometimes) or more on at least 4 of the 7 items. Pre-test Cronbach’s alpha value of the scale was 0.73, and post-test Cronbach’s alpha value was 0.76 [31]. In this study, Cronbach’s alpha coefficients of pre-test, post-test, first, and second follow-up scores were above an acceptable level (Cronbach’s alpha > 0.80).

### Procedure and measurements

The data were collected between December 2023 and June 2024. The intervention was implemented between December 2023 and January 2024. Informed consent forms were sent to parents via adolescents in sealed envelopes. The consent forms were returned to the adolescents by teachers in sealed envelopes within a week. The adolescents whose parents gave consent were informed about the purpose and process of the study and asked to complete the informed consent form.

Adolescents were asked to complete the data collection tools (pre-test) under the researcher’s observation, with the school’s guidance and counselling teacher present. After the evaluation, adolescents who were addicted to digital games were identified. A preparatory session and motivational interviewing sessions were conducted with the adolescents once a week for five weeks by one of the researchers. Following the motivational interviewing, the school’s guidance counsellor teacher applied data collection tools (post-test) (week 6). Following this, the first follow-up (3rd month) in March 2024 and the second follow-up (6th month) in June 2024 were conducted, and the data collection tools were administered to the adolescents by the same school’s guidance and counselling teacher.

### Intervention

Motivational interviewing was administered during the intervention stage of the research. Motivational interviewing consists of two stages; in the first, the four principles (developing discrepancy, empathy, supporting self-efficacy, and resolving resistance) facilitate and cultivate motivation in individuals. The second stage is designed to strengthen the commitment to change [32, 33]. Fidelity to motivational interviewing was ensured through a trained practitioner asking appropriate questions, taking interview notes, encouraging reflection, and using language consistent with the nature of motivational interviewing. All motivational interviewing sessions were delivered by one of the researchers, who had been trained in motivational interviewing and held a Doctor of Philosophy in psychiatric nursing. Motivational interviewing sessions were conducted as follows:

- *Preparation session:* Introductions were made, group rules were established, the group was informed about the purpose and process of the research, expectations were shared, feedback was received, and the session was summarised.
- *Session one (pre-intention):* In this session, the adolescent was prepared for change by raising awareness about internet and digital-game addiction. Adolescents’ behavioural patterns related to using the internet and digital gaming were evaluated. Resistance to situations that make addictive behaviours positive was discussed, and adolescents shared their routines pertaining to internet use and digital games.
- *Session two (intention):* The goal of this session was to share experiences about internet use and digital gaming, to discuss the positive and negative situations affecting addiction, and to reveal the ambivalent feelings of the adolescents.
- *Session three (preparation):* This session was aimed at eliciting positive behavioural change, at discussing the positive and negative effects of behaviours by drawing on past experiences that have encouraged behavioural change, and at creating a commitment to change by making explicit agreements about change.
- *Session four (movement):* The adolescents’ plans to implement behavioural changes were evaluated. Adolescents were encouraged to support their self-efficacy by maintaining their compliance with the target behaviour, focusing on successful actions, feeling intrinsic success, and confirming their self-efficacy assessment.
- *Session five (sustaining):* The goals of the last session were to confirm progress, strengthen the commitment to change, build confidence, reveal high risk situations that may be the precursors of stagnation, identify new strategies and behaviours to overcome these difficulties, maintain belief in change, support competence in change, provide motivation to reinforce change, share adolescents’ achievements and alterations from the interviews, and finally part ways.

### Experimental group

The motivational interviewing, consisting of six sessions (including the preparation session), was conducted by the researcher for the experimental group. The sessions were held on the same day and at the same time every week. Each session lasted an average of 40 min. In this study, motivational interviewing sessions were conducted with nine different groups consisting of five people in the experimental group. One day before each session, adolescents were reminded of the session time by a telephone call. The sessions were conducted in an empty classroom designated by the school administration, with circle seating.

### Control group

The adolescents in the control group did not receive any intervention other than the daily routine practices. Instruments were administered to the adolescents in this group simultaneously with the experimental group session in the 6th week after the intervention. Then, the first follow-up (3rd month) and the second follow-up (6th month) were conducted, and data collection tools were applied. At the end of the study, a webinar or online motivational interviewing on coping skills with internet addiction and digital game addiction was planned for the adolescents. However, no adolescent requested a webinar or motivational interviewing after the study.

### Data analysis

The data were evaluated using IBM SPSS 28.0 (IBM Corp., Armonk, NY). The independent variable was motivational interviewing, and the outcomes were internet addiction and digital game addiction. Descriptive statistics were presented as numbers, percentages, means, and standard deviations (SD). The normality of the data was evaluated using the Shapiro–Wilk test and ± 2 values for kurtosis and skewness. The Chi-square test was used to compare categorical variables. Comparison of binary group data was performed using an independent-samples t-test. To examine the group, time and group*time interaction of the measurements of the experimental and control groups, a two-way mixed-design analysis of variance with repeated measures and a Bonferroni comparison test were used. Missing data in the experimental group were completed using interpolation techniques. Missing data were handled using the multiple imputation method to reduce their impact on the analysis. An intention-to-treat (ITT) analysis was performed to evaluate the data from participants who dropped out of the study. A value of *p* < 0.05 was accepted as the significance level.

### Ethical consideration

Ethical approval was obtained from the Gazi University Ethics Commission (Date and No: 09.05.2023–09; Research Code: 2023-757). Written informed consent was obtained after the parents of the adolescents participating in the study and the adolescents themselves were informed about the research process. The study was conducted in accordance with the principles of the Declaration of Helsinki. The researchers explained to the adolescents that the answers they provided during the study would be used for scientific purposes and that their identity information would remain confidential. Anonymity was well maintained by removing identifiers from the data. The instruments were stored in a locked cabinet, and the collected responses were protected by encryption on the computer.

## Results

**Fig. 1:**
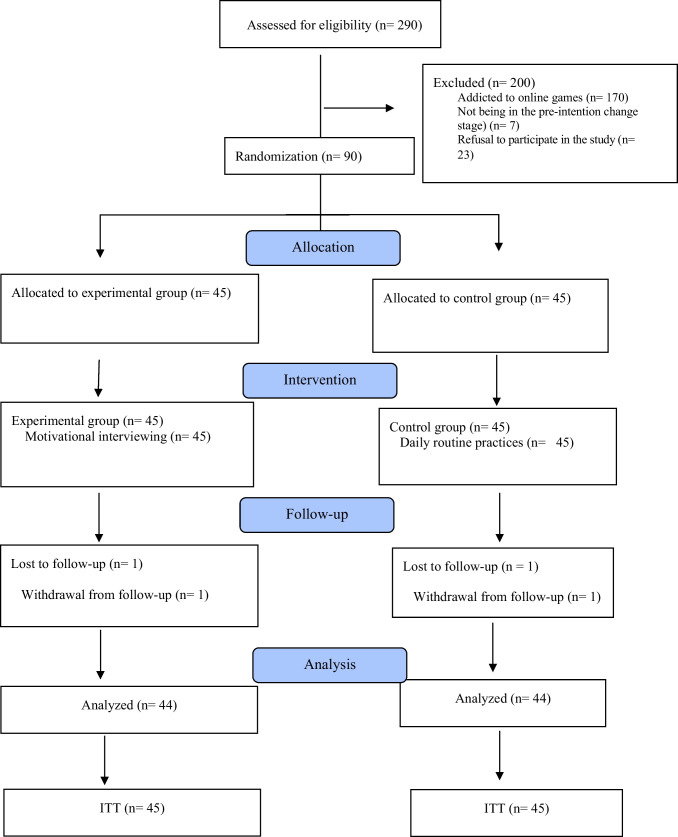
Consort flowchart

Of the recruited adolescents (*n* = 90), one participant from the experimental group and one from the control group voluntarily left the study. The study was completed with 88 adolescents; 44 in the experimental group and 44 in the control group (Fig. 1). In the post hoc power analysis, the study power was 0.99 with an alpha of 0.05 and a 95% confidence interval.

The findings regarding the individual characteristics of the adolescents in the experimental and control groups are presented in Table 1. There was no statistically significant difference between the experimental and control groups in individual characteristics (*p* > 0.05).

**Table 1:** Individual characteristics of the adolescents

Individual characteristics	Experimental group (*n* = 44)		Control group (*n* = 44)		Statistical analysis
Age (Mean, SD)	14.11 (0.32)		14.07 (0.25)		*t* = 0.735 *p* = 0.464
Frequencies					
Sex	*n*	%	*n*	%	
Girl	22	50.0	22	50.0	*χ*^2^ = 0.000
Boy	22	50.0	22	50.0	*p* = 1.000
Maternal education level					
Primary/secondary	10	22.7	7	15.9	*χ*^2^ = 0.656
High school/university	34	77.3	37	84.1	*p* = 0.418
Paternal education					
Primary/secondary	3	6.8	2	4.5	*χ*^2^ = 0.212
High school/university	41	93.2	42	95.5	*p* = 0.645
Family income					
Equal to expenses	36	81.8	36	81.8	*χ*^2^ = 0.000
More than expenses	8	18.2	8	18.2	*p* = 1.000
Academic achievement					
Moderate	34	77.3	36	81.8	*χ*^2^ = 0.279
Good	10	22.7	8	18.2	*p* = 0.597
Relationship with family					
Moderate	12	27.3	6	13.6	*χ*^2^ = 2.514
Good	32	72.7	38	86.4	*p* = 0.113
Relationship with friends					
Moderate	33	75.0	38	86.4	*χ*^2^ = 1.823
Good	11	25.0	6	13.6	*p* = 0.177
Daily internet use time					
≤ 2 h	13	29.5	8	18.2	*χ*^2^ = 3.346
3–4 h	13	29.5	21	47.7	*p* = 0.188
5–6 h	18	40.9	15	34.1	
Daily online gaming time					
≤ 2 h	13	29.6	9	20.5	*χ*^2^ = 1.148
3–4 h	17	38.6	21	47.7	*p* = 0.563
5–6 h	14	31.8	14	31.8	
Owning of smartphone					
1–2 y	7	15.9	7	15.9	*χ*^2^ = 0.237
3 years	12	27.3	14	31.8	*p* = 0.888
4–6 years	25	56.8	23	52.3	

*SD* standard deviation, *χ*^*2*^ chi-square test, *t* independent *t*-test

The two-way mixed design ANOVA revealed significant differences in internet addiction scores between groups (experimental vs. control) (*F* (1) = 188.956, *p* < 0.001), time (*F* (1.62) = 279.328, *p* < 0.001), and the group-by-time interaction effect (*F* (1.62) = 354.176, *p* < 0.001). It was determined that the mean scores of internet addiction after motivational interviewing in the experimental group decreased significantly compared to the control group (*p* < 0.001), thus confirming the hypothesis (H_1_). The intervention had a large effect size (*η*^2^ = 0.805) and high power (99.9%) (Table 2).

**Table 2:** Internet addiction and digital game addiction mean scores of the experimental and control groups and two-way mixed design analysis of variance results

Scales and measurements	Experimental group (*n* = 44)	Control group (*n* = 44)		Group	Time	Group × time interaction
	Mean (SD)	Mean (SD)				
Internet addiction						
Pre-test	53.68 (5.17)^A,a^	53.23 (5.30)^A,a^	*F*	188.956	279.328	354.176
Post-test	37.95 (4.45)^A,,b^	53.75 (5.03)^B,a^	*p*	< 0.001	< 0.001	< 0.001
First follow up	35.43 (4.93)^A,c^	54.38 (5.14)^B,a^	*η*^2^	0.687	0.765	0.805
Second follow up	35.04 (4.69)^A,c^	54.45 (5.30)^B,a^				
Digital game addiction						
Pre-test	31.70 (3.45)^A,a^	31.45 (2.72)^A,a^	*F*	261.164	234.956	282.983
Post-test	20.63 (2.51)^A,b^	31.72 (3.15)^B,a^	*p*	< 0.001	< 0.001	< 0.001
First follow up	19.11 (3.01)^A,c^	32.27 (3.18)^B,a^	*η*^2^	0.752	0.732	0.767
Second follow up	18.61 (3.20)^A,c^	31.98 (3.66)^B,a^				

*F* two-way mixed design analysis of variance, *η*^*2*^ partial eta squared, *SD* standard deviation

^A,B^Different capital letters on the same row indicate the differences between experimental and control groups

^a,b,c^Different lowercase letters in the same column indicate the differences between measurements

The two-way mixed design ANOVA discovered significant differences in digital game addiction scores between the groups (experimental vs. control) (*F* (1) = 261.164, *p* < 0.001), time (*F* (1,71) = 234.956, *p* < 0.001), and the group-by-time interaction effect (*F* (1.71) = 282.983, *p* < 0.001). The mean scores of digital game addiction after the motivational interviewing in the experimental group decreased significantly compared to the control group (*p* < 0.001), thus verifying the hypothesis (H_2_). The intervention had a large effect size (*η*2 = 0.767) and high power (99.9%). As a result of the ITT (*n* = 90) analysis conducted to determine the effect of adolescents who left the study, the findings obtained with ITT and per-protocol were similar.

## Discussion

There is an increasing need for effective, evidence-based intervention programs to combat addictive behaviours, driven by rapid technological developments and the ease of internet access [34, 35]. The present study revealed that the level of internet addiction and digital game addiction of the experimental group decreased significantly after motivational interviewing in comparison to the control group. These findings show that motivational interviewing, when used as a practical method in combating addiction, is effective in providing behavioural change, especially in adolescents who are ambivalent or reluctant to change. This research suggests that motivational interviewing sessions, using techniques such as open-ended questioning, affirmations, reflective listening, summarising, and decision-making balance, help adolescents uncover and resolve indecision or contradictions in their behaviour, thereby increasing intrinsic motivation. Additionally, it is believed that encouraging adolescents to participate in diverse activities through assignments and face-to-face social activity plans leads to behavioural change and increased motivation to maintain healthy digital behaviours.

In the literature, similar results were obtained in a limited number of studies examining the effect of motivational interviewing in combating internet addiction and digital game addiction [23, 25]. In addition, multiple interventions, including cognitive-behavioural therapy and family-based psychological approaches [36] and health education [26], in conjunction with motivational interviewing, have produced positive results in preventing digital game addiction. Empirically, the motivational interviewing approach used to reduce and prevent addiction-related behaviours can reveal an individual’s intrinsic motivation [23, 25]. In this study, a significant decrease occurred in addiction-related behaviours in adolescents compared to the control group by providing intrinsic motivation and supporting adolescents to resolve their ambivalent feelings and reveal their reasons for change through motivational interviewing. At the same time, adolescents who were reluctant and resistant regarding behavioural change were enabled to reach their individual goals related to reducing addictive behaviours using their impetus for the transition from the pre-intention stage to the maintenance stage according to the change stage assessment form. It is thought that planned and regularly applied motivational interviewing is an effective method in mitigating symptoms of internet addiction and digital game addiction behaviours in adolescents.

Since these circumstances increase the risk of digital game addiction in adolescents, effective intervention programs related to digital game addiction in adolescents are of utmost importance [25]. The findings of this study show that motivational interviewing encourages positive behaviours related to digital game addiction in adolescents. The authors believe that motivational interviewing principles, including empathy, revealing inconsistencies, reducing resistance, and strengthening self-efficacy, when implemented in sessions, may guide adolescents towards healthy digital behaviours. Given that digital game addiction, which is associated with stress, poor psychological functioning, and low self-esteem and negatively affects the personal well-being of individuals, is considered a current public health problem [7, 25, 37], motivational interviewing may be valuable as an approach to prevent digital game addiction.

There are some limitations. The data were collected using self-reporting techniques, which may have affected the quality of the data obtained. In addition, the researcher’s presence during pre-test data collection may have affected adolescents’ responses and comfort, perhaps introducing external pressure that could have biased their feedback. Although the digital game addiction scale used in this study was valid and reliable, it may not be as satisfactory as a clinical diagnosis. School administrators did not approve audio recording; therefore, it could not be used for fidelity assessment of the motivational interviewing sessions. Finally, the passive control group may have led to the observed effects being greater than the intervention effect, and to non-specific effects being uncontrollable. Future studies could incorporate active comparison groups and clinically diagnostic inclusion criteria to strengthen methodological rigour.

## Conclusion

This randomised controlled study suggests that motivational interviewing may be associated with reductions in mitigating symptoms of internet and digital game addiction in adolescents, and may contribute to positive behavioural change. Although this study employed a randomised controlled design, the findings should be interpreted with caution due to limitations, including the use of a passive control group, diagnostic criteria based on the Digital Game Addiction Scale, and the lack of audio recording. Strategies that include motivational interviewing in the fight against internet and digital game addiction should be developed. In the future, experimental studies with active comparison groups are needed to observe the superiority of various interventions for the prevention of addictive behaviours in adolescents.

## Data Availability

The data for this study are available from the corresponding author upon reasonable request.
